# Comparative Analysis of Cartilage Conduction Hearing Aid Users and Non-Users: An Investigative Study

**DOI:** 10.3390/audiolres13040049

**Published:** 2023-07-27

**Authors:** Satofumi Sugimoto, Tadao Yoshida, Yukari Fukunaga, Aya Motegi, Ken Saito, Masumi Kobayashi, Michihiko Sone

**Affiliations:** 1 Department of Otorhinolaryngology, Nagoya University Graduate School of Medicine, 65 Tsurumai-cho, Showa-ku, Nagoya 466-8550, Japan; tadaoy@med.nagoya-u.ac.jp (T.Y.); motegi.aya@med.nagoya-u.ac.jp (A.M.); saisaisai425@med.nagoya-u.ac.jp (K.S.); masumi@med.nagoya-u.ac.jp (M.K.); michsone@med.nagoya-u.ac.jp (M.S.); 2 Department of Rehabilitation, Nagoya University Graduate School of Medicine, 65 Tsurumai-cho, Showa-ku, Nagoya 466-8550, Japan; fukunay@med.nagoya-u.ac.jp

**Keywords:** cartilage conduction hearing aid, aural atresia, meatal stenosis, bone conduction hearing aids, conductive hearing loss

## Abstract

Clinical findings on cartilage conduction hearing aids (CCHAs) have gradually become clear; however, few reports include a large number of cases. This study included 91 ears from 69 patients who underwent CCHA fitting in our hospital. Their ears were divided into six groups (i.e., bilateral aural atresia or severe canal stenosis, unilateral aural atresia or severe canal stenosis, chronic otitis media or chronic otitis externa with otorrhea, sensorineural hearing loss, mixed hearing loss, and conductive hearing loss) according to their clinical diagnosis and type of hearing loss. Most clinical diagnoses were aural atresia or meatal stenosis (bilateral, 21.8%; unilateral, 39.6%). The purchase rate of CCHAs was higher in the closed-ear group (bilateral, 77.3%; unilateral, 62.5%). In the bilateral closed-ear group, air conduction thresholds at 1000, 2000, and 4000 Hz and aided thresholds with CCHAs at 4000 Hz were significantly lower in the purchase group than the non-purchase group. No significant difference was observed between the purchase and non-purchase groups in the unilateral closed-ear group. In the bilateral closed-ear group, air conduction thresholds and aided thresholds were associated with the purchase rate of CCHAs. In the unilateral closed-ear group, factors other than hearing might have affected the purchase rate of CCHAs.

## 1. Introduction

Cartilage conduction hearing aids (CCHAs) are a new type of hearing aid. Hosoi found that an unmistakable sound can be heard when a vibration signal is delivered to the auricular cartilage using a transducer, a process which was termed “cartilage conduction” [1]. Hosoi and Nishimura’s group continued their cartilage conduction research [2,3,4,5] and developed a CCHA [6,7,8]. The prototype CCHA was first reported in 2010 [6]. Body-aid [7] and behind-the-ear [8] types were developed shortly afterwards. To fix the transducers of the CCHAs, a double-sided skin tape and ear chip were used. A double-sided skin fixation is available for all ear conditions. On the one hand, ear chip fixation is only employed in select cases, but is useful for improving comfort. Acoustic devices that utilize cartilage conduction, including earphones and smartphones, have also been developed [4]. CCHAs that can adjust their volume depending on the frequency are most desirable for patients with hearing loss. CCHAs have the advantages of comfort, stable fixation, aesthetics, and non-invasiveness [9,10]. Based on the characteristic mechanism of cartilage conduction, CCHAs provide benefits for patients with aural atresia and chronic otitis media, with which it is difficult to use air conduction hearing aids (ACHAs), and these patients require bone conduction hearing aids (BCHAs) [6,7,10,11]. CCHAs have been used clinically in Japan since November 2017, and the clinical characteristics of CCHAs have been reported [12,13,14,15,16,17,18,19,20]. Nishiyama et al. investigated child and adult candidates for CCHA treatment separately and reported that patients with ear canal stenosis or aural atresia were the most suitable candidates [12,13]. Sakamoto et al. evaluated the benefits of CCHA in patients with unilateral congenital aural atresia and reported that their speech recognition scores improved in noisy environments [14]. Komune et al. investigated patients after lateral temporal bone resection and reported upon the availability of CCHAs for postoperative hearing compensation [15]. To investigate the clinical use of CCHAs in Japan, we conducted a survey of nine medical institutions with 256 patients who had tried CCHAs [16]. The survey reported a high purchase rate in ears with aural atresia or severe canal stenosis. In addition, a high purchase rate was also reported among patients with refractory continuous otorrhea who experienced difficulties with ACHAs. In this way, clinical findings on CCHAs have gradually become clear; however, there are few reports with a large number of cases. In this study, we investigated cases with CCHAs in our hospital and identified some ways to improve CCHA fitting.

## 2. Materials and Methods

### 2.1. Participants

The study included 91 ears from 69 patients (35 men and 34 women; age range, 2–83 years) who underwent CCHA fitting in our university hospital between December 2017 and December 2022. To examine the effect of CCHAs on closed ears clearly in this study, we excluded ears on which meatoplasty had been performed. Cases with aural atresia or severe canal stenosis were recruited into bilateral or unilateral closed-ear groups. Cases with chronic otitis media or chronic otitis externa with otorrhea who experienced difficulties with ACHAs were recruited into the continuous otorrhea group. Other diseases were divided into three groups (i.e., sensorineural hearing loss, mixed hearing loss, and conductive hearing loss) according to the type of hearing loss.

### 2.2. Audiometry

Pure-tone audiometry was performed on patients aged 6 and older using an AA-78 diagnostic audiometer (Rion Co. Ltd., Tokyo, Japan). Behavioral audiometry was performed on patients aged 2 to 5 using an AA-73 diagnostic audiometer (Rion). These tests were performed in a soundproof compartment, primarily on the patient’s first visit to our hospital. Air and bone conduction audiometric measurement thresholds were calculated for each ear at 250, 500, 1000, 2000, and 4000 Hz. The air and bone conduction threshold averages were calculated using five averages (250, 500, 1000, 2000, and 4000 Hz thresholds).

### 2.3. CCHA Fitting and Evaluations

HB-J1CC and HB-A2CC CCHAs (Rion) were used for CCHA fitting. When patients tested the CCHAs, a double-sided skin tape (#1522; 3M Japan Limited, Tokyo, Japan) was used to fix the transducers to the tragal area, which consists mostly of cartilage. The position of the transducers was similar to that of previous reports [10,16,17]. Aided thresholds at 250, 500, 1000, 2000, and 4000 Hz were measured via sound field tests using an AA-76 diagnostic audiometer (Rion) in a soundproof compartment in all patients, primarily at the time of first fitting. The aided threshold average was calculated using five averages (250, 500, 1000, 2000, and 4000 Hz thresholds). The patients brought CCHAs home and tested them for at least two weeks. The patients then decided whether to purchase CCHAs. They were asked why they purchased or did not purchase CCHAs. Ear impressions were taken during the purchase stage if ear-chip-type transducers were available.

### 2.4. Ethics Review

This study was approved by the Ethics Review Committee of the Nagoya University School of Medicine, Nagoya, Japan (No. 2022-0492).

### 2.5. Statistical Analyses

The IBM SPSS Statistics software (version 28, IBM Corp., Armonk, NY, USA) was used for statistical analyses. The significance level was set to 5%. The sex distribution and presence of previous hearing aids were compared between the two groups using the *X*^2^ test. Air and bone conduction thresholds, aided thresholds with CCHAs, and mean ages were compared between CCHA purchase and non-purchase cases using the Mann–Whitney U test. Comparisons between aided thresholds with previous hearing aids and those with CCHAs were also assessed using the Mann–Whitney U test.

## 3. Results

Table 1 shows the demographic and clinical data for ears fitted with CCHAs. Most clinical diagnoses were aural atresia or meatal stenosis (62 ears, 61.4%), and there were more unilateral cases (40 ears, 39.6%) than bilateral cases (22 ears, 21.8%). The bilateral closed-ear group included 18 cases with congenital aural atresia or meatal stenosis, 2 cases with acquired fibrotic aural atresia caused by chronic irritation and inflammation, and 2 cases with congenital meatal stenosis with chronic inflammation. The average air conduction threshold in all ears was 63.1 dB. All cases had bilateral hearing loss. The unilateral closed-ear group included 36 cases with congenital aural atresia or meatal stenosis and 4 cases with acquired fibrotic aural atresia caused by carcinoma operations in the ear canal or chronic irritation and inflammation. The average air conduction threshold in all opposite ears was 17.3 dB. There were four opposite ears with average air conduction thresholds of more than 30 dB. The continuous otorrhea group included five cases. Most cases were affected bilaterally. All cases had bilateral hearing loss. The variation in bone conduction hearing was small, including in the ipsilateral and contralateral ears. The sensorineural hearing loss group included three cases. The average air conduction threshold in all opposite ears was 46.3 dB. There was an opposite ear with normal hearing and another two ears with average air conduction thresholds of more than 50 dB. The mixed hearing loss group included 16 cases. The average air conduction threshold in all opposite ears was 61.4 dB. There were two opposite ears with normal hearing and another fourteen ears with average air conduction thresholds of more than 30 dB. The conductive hearing loss group included five cases. Most cases were affected bilaterally. All cases had bilateral hearing loss.

Patients bought CCHAs for a total of 47 ears. Forty-two of these ears suffered from aural atresia or severe canal stenosis (purchase rate in bilateral cases, 77.3%; purchase rate in unilateral cases, 62.5%). The remainder included an ear with chronic otitis externa with otorrhea (purchase rate, 20.0%), an ear with mixed hearing loss (purchase rate, 6.3%), and three ears with conductive hearing loss (purchase rate, 60.0%). The purchase rate for sensorineural hearing loss was 0%. In the unilateral closed-ear group, patients whose opposite ear’s hearing level was more than 30 dB had a higher purchase rate than those whose opposite ear’s hearing level was less than 30 dB. (100% vs. 58.3%). In the four purchase cases with mixed or conductive hearing loss, all four opposite ears suffered from conductive hearing loss. Three patients chose CCHAs for cosmetic reasons and refused to try the ordinary behind-the-ear type of ACHA. Another patient, whose opposite ear had severe canal stenosis, chose CCHAs because he wanted to use the same hearing aids bilaterally. We focused on the closed-ear group because both the number of ears and the purchase rate were high.

**Figure 1:**
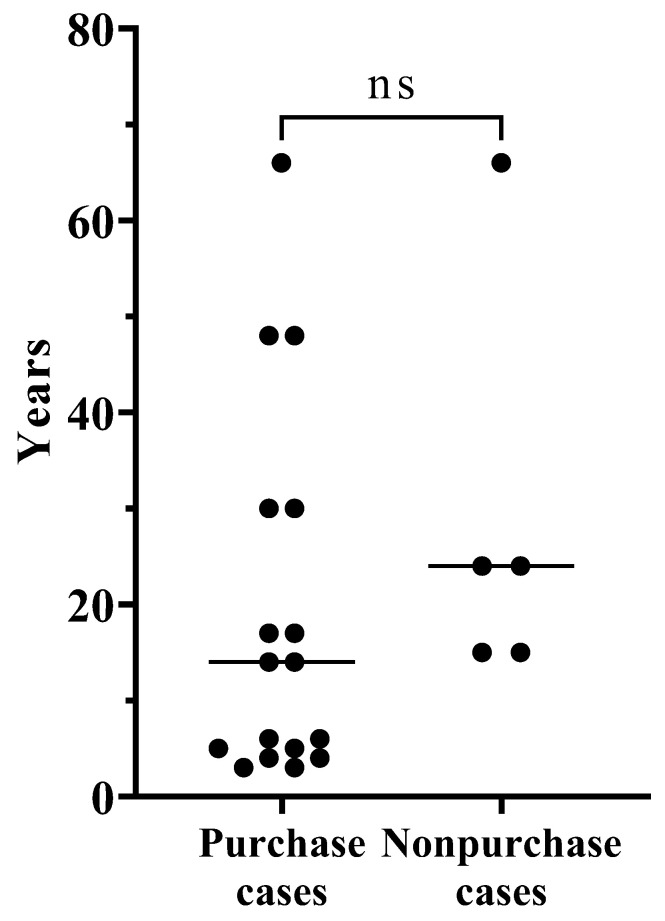
The age distribution between CCHA purchase and non-purchase cases in bilateral aural atresia or severe canal stenosis.

Table 2 shows comparisons between the CCHA purchase and non-purchase cases in bilateral aural atresia or severe canal stenosis. The mean age was younger in the purchase group; however, no significant difference was observed. The age distribution is shown in Figure 1. For all cases aged 6 years and younger, CCHAs were purchased. Many ears had been fitted with hearing aids, all of which were BCHAs. The number of patients with a history hearing aids and the sex distribution between the two groups did not differ significantly. Air conduction thresholds were lower at all frequencies in the purchase group, for which there was a significant difference at 1000, 2000, and 4000 Hz, but there was no significant difference in the bone conduction thresholds. Aided thresholds with CCHAs were lower in the purchase group, except at 250 Hz, and a significant difference was found at 4000 Hz. When comparing aided thresholds with previous hearing aids and those with CCHAs in each group, the aided thresholds with CCHAs were slightly higher in both groups at many frequencies. There was a significant difference in the non-purchase group at 1000 Hz.

**Figure 2:**
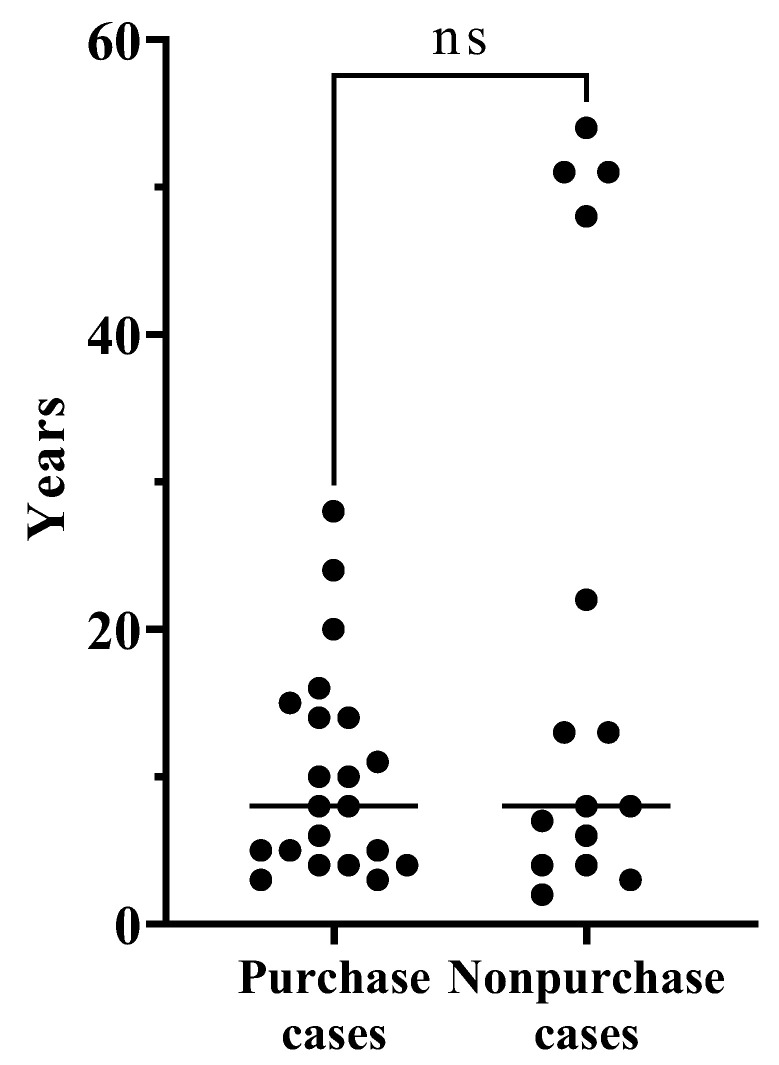
The age distribution between CCHA purchase and non-purchase cases in unilateral aural atresia or severe canal stenosis in which the opposite ear’s hearing level was less than 30 dB.

To observe the effect of CCHAs on unilateral aural atresia or severe canal stenosis clearly, we performed a comparison between CCHA purchase and non-purchase cases in unilateral closed ears whose opposite ear’s hearing level was less than 30 dB (Table 3). The mean age was younger in the purchase group; however, no significant difference was observed. The age distribution is shown in Figure 2. No patients over 47 years old purchased CCHAs. Since the opposite ear’s hearing level was less than 30 dB in all cases, no patients were fitted with hearing aids before their first visit to our hospital. The number of patients with a history of hearing aids and the sex distribution between the two groups did not differ significantly. Although affected ears, as well as the contralateral ears, were examined, no significant difference was observed between the two groups when their hearing was compared. There was no significant difference in the aided thresholds for CCHAs.

## 4. Discussion

In this study, we investigated 91 ears that underwent CCHA fitting in our hospital. The number of ears and the purchase rate were high in the closed-ear group, as reported previously [16,17].

Patients with bilateral closed ears usually require hearing aids [21,22,23]. The percentage of previous hearing aid users in the bilateral closed-ear group was highest in the six groups. Regarding the bilateral closed-ear group, purchase cases showed significantly lower aided thresholds with CCHAs at 250 and 500 kHz than the non-purchase cases [17]. Our study found that aided thresholds at 4000 Hz were significantly lower in the purchase group. Overall, better aided thresholds with CCHAs contribute to CCHA purchases. Many cases with bilateral closed ears had been fitted with BCHAs. The patients compared the new CCHAs with their current BCHAs and decided whether to purchase the CCHAs. The aided thresholds with CCHAs in the bilateral closed-ear group were relatively good; therefore, most patients bought CCHAs. The BCHA transducer is relatively big and fixed with a headband or a similar device; therefore, BCHAs are more visible than CCHAs and ACHAs. If BCHCs are to function well, the transducer must be pushed tightly against the head. However, continued use can cause pain, irritation, and discomfort [24,25]. Meanwhile, BCHAs have the advantage of being easy to put on and take off. Most closed-ear cases cannot use ear chips and must use a double-sided skin tape to fix CCHA transducers, which makes it difficult to attach or remove CCHAs. Even after purchasing CCHAs with appropriate aided thresholds, some patients with bilateral closed ears continued to use BCHAs in combination with them, mainly in situations where it was necessary to put the hearing aid on and take it off easily. Parents in the bilateral closed-ear group often had a strong desire to improve the appearance of their children, as well as their hearing. Many of them decided in advance to purchase CCHAs for their children before visiting a doctor. If a patient’s aided threshold with CCHAs is poor compared to with BCHAs, we must propose that parents make their purchasing decisions carefully, especially for young children who cannot express their own opinion about which aid is better.

Patients with unilateral closed ears often have another ear with normal hearing and do not consider hearing aids to be essential. On the other hand, the negative effect of unilateral severe hearing loss on communication, development, and education has been reported [26,27], and hearing aids for the affected ear are desirable. In the unilateral closed-ear group, in which the opposite ear’s hearing level was normal, it was reported that purchase cases were significantly younger than non-purchase cases and no obvious differences were observed in both aided and unaided thresholds [17]. The mean age was also younger in the purchase group in our study; however, no significant difference was observed. Most patients with unilateral closed ears were under 30 years old. Meanwhile, the other four middle-aged patients did not purchase CCHAs. These four cases appeared to raise the mean age of the non-purchasing group. We compared these four middle-aged cases with the other young candidates. However, no difference was observed in air and bone conduction thresholds, aided thresholds with CCHA, or reasons for not purchasing CCHAs (itchiness, noise, and annoyances associated with using CCHA). For middle-aged candidates in the unilateral closed-ear group, the discomfort of wearing CCHAs might have outweighed the benefits of reducing the left–right difference in hearing. On the other hand, in the unilateral closed-ear group, in which the opposite ear’s hearing level was more than 30 dB, the need for hearing aids was considered higher than in the group with unilateral hearing loss.

The purchase rate in the continuous-otorrhea group was lower than that reported previously [16,17]. All our cases with continuous otorrhea were over 70 years old. Due to age-related hearing loss, it is plausible that a decrease in the purchase rate occurred because the adequate aided thresholds were not reached. The conductive hearing loss group showed a high purchase rate similar to the closed-ear group; however, there were only five ears in the conductive hearing loss group, and CCHAs were mainly purchased for them because the patients considered CCHAs to look better than the ordinary behind-the-ear type of ACHAs. This purchase rate may be overestimated, and further investigations are required. In this study, few patients with sensorineural or mixed hearing loss chose to purchase CCHAs. Meanwhile, more than 36% of patients purchased CCHAs in relatively similar groups in previous studies [12,16,17]. The purchase rate might have deteriorated because we provided an opportunity to compare CCHAs with ACHAs, which are often less expensive and usually have higher acoustic gain than CCHAs. ACHAs appear to be suitable for patients with sensorineural or mixed hearing loss. In the study, when patients tested the CCHAs, a double-sided skin tape was used to fix the transducers. If ear impressions had been taken in advance and ear-chip-type transducers were available at the time of CCHA fitting, the purchase rate might have been higher. Using the ear chip increases the transducer stability of CCHAs and allows patients to put CCHAs on and take them off easily. Ear-chip-type fixation is recommended when its insertion is enabled by the placement of the fixation [17]. However, a double-sided skin tape fixation for CCHA fitting has advantages. This fixation method is available for all ear conditions and reduces the time required for fitting and unnecessary ear chip costs [17].

It is necessary to consider the possibility that hearing aid prices influence hearing aid purchases. One HB-J1CC CCHA costs JPY 300,000, and two HB-J1CC CCHAs cost JPY 510,000. One HB-A2CC CCHA costs JPY 350,000, and two HB-A2CCs cost JPY 600,000. In this study, most previously used BCHAs were Mini Digital BCHAs (Starkey Hearing Technologies, Minnesota, MN, USA) fixed with hard headbands. One Mini Digital BCHA costs JPY 189,000, and two Mini Digital BCHAs cost JPY 346,000. ACHAs range from cheap to expensive, but some behind-the-ear types of ACHAs can be purchased for JPY 100,000 to JPY 200,000 per unit. CCHAs and some behind-the-ear types of ACHAs are sold at special prices for those under 20 years of age. In Japan, one HB-J1CC CCHA costs JPY 150,000, and two HB-J1CC CCHAs cost JPY 300,000. One HB-A2CC CCHA costs JPY 175,000, and two HB-A2CCs cost JPY 350,000. Some behind-the-ear types of ACHAs cost approximately JPY 43,900 in special cases. Meanwhile, Mini Digital BCHAs are sold at the same price regardless of age. These prices can be summarized as follows: for those over 20, ACHAs and BCHAs are cheaper than CCHAs. For those under 20, ACHAs are also cheaper than CCHAs. However, the price of BCHAs is not much different from that of CCHAs. In this study, two-thirds of patients who tested CCHAs were under 20, and those who used BCHAs might have been more likely to choose CCHAs from an economic point of view.

There are some limitations to the present study. The purchase rate could be influenced not only by the attainment of suitable aided thresholds, but also by considerations of aesthetics, comfort, and the economic dimension. The purchase rate is not a pure measure of CCHA effectiveness. Meanwhile, when considering which patients should be recommended for CCHAs, it is good to focus on the purchase rate, as their appearance, comfort and economic advantages are also considered when they are compared with BCHAs, which are often competitive. Due to the examination taking place in only one facility, regional factors, such as subsidies by local governments, might have affected our results. We used data from a relatively early sales stage; therefore, purchase trends may change in the future. Further investigations are required.

## 5. Conclusions

The purchase rate of CCHAs was particularly high in ears with aural atresia or severe canal stenosis. In the bilateral closed-ear group, air conduction thresholds and aided thresholds were associated with the purchase of CCHAs. In the unilateral closed-ear group, factors other than hearing might affect the purchase of CCHAs.

## Figures and Tables

**Table 1:** Demographic and clinical data for ears fitted with CCHAs.

Group	n	Age (Year)	Sex	Previous Hearing Aids	Air Conduction Thresholds (dB HL)	Bone Conduction Thresholds (dB HL)	Aided Thresholds CCHA (dB HL)	Purchase Rate CCHA
		Average (SD)	(Female /Male)	(No/ACHA /BCHA)	Average (SD)	Average (SD)	Average (SD)	
Bilateral aural atresia or severe canal stenosis	22	21.1 (12.2)	12/10	6/0/16	63.1 (12.2)	9.9 (6.5)	33.0 (3.2)	77.3%
Unilateral aural atresia or severe canal stenosis	40	13.7 (13.9)	20/20	39/0/1	70.6 (9.5)	9.0 (7.7)	38.6 (6.5)	62.5%
Chronic otitis media or chronic otitis externa with otorrhea	5	74.2 (4.5)	5/0	0/5/0	65.2 (14.0)	43.6 (3.4)	44.3 (7.5)	20.0%
Sensorineural hearing loss	3	64.7 (20.3)	0/3	1/2/0	58.3 (8.4)	53.3 (9.5)	45.0 (4.2)	0.0%
Mixed hearing loss	16	38.3 (30.4)	7/9	8/6/2	73.8 (12.9)	27.6 (14.4)	42.6 (6.8)	6.3%
Conductive hearing loss	5	9.8 (3.4)	2/3	2/1/2	48.8 (13.4)	1.4 (2.2)	32.0 (2.9)	60.0%
Total	91	24.6 (25.3)	46/45	56/14/21	67.5 (12.9)	15.6 (15.3)	37.9 (6.9)	51.6%

ACHA, air conduction hearing aid; BCHA, bone conduction hearing aid; CCHA, cartilage conduction hearing aid; SD, standard deviation.

**Table 2:** Comparison between CCHA purchase cases and non-purchase cases in bilateral aural atresia or severe canal stenosis.

	Purchase (*n* = 17)	Non-Purchase (*n* = 5)
	Average (SD)	Average (SD)
Age (Year)	18.8 (18.6)	28.8 (19.0)
Sex (Female/Male)	9/8	3/2
Previous hearing aids (No/BCHA)	5/12	1/4
Air conduction thresholds (dB HL)		
250 Hz	67.9 (20.1)	81.0 (5.8)
500 Hz	65.9 (19.3)	83.0 (8.7)
1000 Hz *	60.0 (13.9)	71.0 (5.8)
2000 Hz *	54.1 (7.7)	64.0 (5.8)
4000 Hz *	52.6 (13.0)	68.0 (8.7)
Bone conduction thresholds (dB HL)		
250 Hz	5.0 (8.6)	3.0 (6.8)
500 Hz	5.0 (8.9)	1.0 (4.9)
1000 Hz	13.2 (12.0)	6.0 (8.6)
2000 Hz	20.3 (11.0)	26.0 (7.3)
4000 Hz	7.1 (7.1)	9.0 (12.4)
Aided thresholds with CCHA (dB HL)		
250 Hz	37.9 (5.2)	33.0 (2.4)
500 Hz	33.5 (5.1)	34.0 (2.0)
1000 Hz	27.6 (4.6)	30.0 (0.0) *
2000 Hz	31.2 (5.3)	33.0 (2.4)
4000 Hz *	32.1 (9.4)	45.0 (11.0)
Aided thresholds with previous hearing aids (dB HL)		
250 Hz	37.8 (16.3)	47.5 (17.5)
500 Hz	33.3 (15.6)	30.0 (5.0)
1000 Hz	28.3 (7.8)	22.5 (2.5) *
2000 Hz	27.2 (7.9)	30.0 (10.0)
4000 Hz	33.2 (8.9)	37.5 (7.5)

BCHA, bone conduction hearing aid; CCHA, cartilage conduction hearing aid; SD, standard deviation; * *p* < 0.05.

**Table 3:** Comparison between CCHA purchase cases and non-purchase cases in unilateral aural atresia or severe canal stenosis in which the opposite ear’s hearing level was less than 30 dB.

	Purchase (*n* = 21)	Non-Purchase (*n* = 15)
	Average (SD)	Average (SD)
Age (Year)	10.3 (7.0)	19.6 (19.6)
Sex (Female/Male)	11/10	8/7
Previous hearing aids (No/Yes)	21/0	15/0
Ears with atresia auris or meatal stenosis		
Air conduction thresholds (dB HL)		
250 Hz	85.7 (11.5)	81.0 (10.7)
500 Hz	78.3 (12.6)	78.7 (11.9)
1000 Hz	67.1 (13.1)	70.7 (10.9)
2000 Hz	61.0 (11.5)	67.0 (14.2)
4000 Hz	61.2 (14.1)	64.3 (14.9)
Bone conduction thresholds (dB HL)		
250 Hz	5.6 (11.0)	5.0 (8.0)
500 Hz	7.1 (15.5)	6.4 (7.2)
1000 Hz	7.9 (10.8)	10.7 (4.9)
2000 Hz	16.3 (10.2)	16.1 (8.7)
4000 Hz	5.3 (10.1)	9.6 (12.0)
Aided thresholds with CCHAs (dB HL)		
250 Hz	51.8 (14.6)	42.1 (12.3)
500 Hz	39.3 (7.6)	41.1 (8.7)
1000 Hz	31.9 (5.5)	34.6 (4.0)
2000 Hz	33.8 (6.5)	35.7 (5.9)
4000 Hz	36.2 (8.7)	42.9 (11.3)
Unaffected ears		
Air conduction thresholds (dB HL)		
250 Hz	18.8 (8.2)	17.7 (6.5)
500 Hz	15.7 (8.6)	13.0 (7.7)
1000 Hz	11.7 (6.8)	11.0 (8.2)
2000 Hz	10.2 (7.0)	9.7 (9.4)
4000 Hz	8.8 (7.9)	12.0 (8.1)
Bone conduction thresholds (dB HL)		
250 Hz	12.3 (6.4)	13.9 (5.7)
500 Hz	9.6 (8.9)	10.0 (5.3)
1000 Hz	8.1 (5.7)	12.8 (5.3)
2000 Hz	13.5 (7.4)	10.6 (5.0)
4000 Hz	5.0 (10.4)	6.7 (10.5)

CCHA, cartilage conduction hearing aid; SD, standard deviation.

## Data Availability

The data used to support the findings of this study are available within the article.
